# Soluble T Cell Receptor Vβ Domains Engineered for High-Affinity Binding to Staphylococcal or Streptococcal Superantigens 

**DOI:** 10.3390/toxins6020556

**Published:** 2014-01-27

**Authors:** Preeti Sharma, Ningyan Wang, David M. Kranz

**Affiliations:** Department of Biochemistry, University of Illinois, Urbana, IL 61801, USA; E-Mails: sharma39@illinois.edu (P.S.); ningyan.wang@gmail.com (N.W.)

**Keywords:** superantigens, yeast display, directed evolution, affinity maturation, T-cell receptor, complementarity determining regions, framework regions

## Abstract

*Staphylococcus aureus* and group A *Streptococcus* secrete a collection of toxins called superantigens (SAgs), so-called because they stimulate a large fraction of an individual’s T cells. One consequence of this hyperactivity is massive cytokine release leading to severe tissue inflammation and, in some cases, systemic organ failure and death. The molecular basis of action involves the binding of the SAg to both a T cell receptor (TCR) on a T cell and a class II product of the major histocompatibility complex (MHC) on an antigen presenting cell. This cross-linking leads to aggregation of the TCR complex and signaling. A common feature of SAgs is that they bind with relatively low affinity to the variable region (V) of the beta chain of the TCR. Despite this low affinity binding, SAgs are very potent, as each T cell requires only a small fraction of their receptors to be bound in order to trigger cytokine release. To develop high-affinity agents that could neutralize the activity of SAgs, and facilitate the development of detection assays, soluble forms of the Vβ regions have been engineered to affinities that are up to 3 million-fold higher for the SAg. Over the past decade, six different Vβ regions against SAgs from *S. aureus* (SEA, SEB, SEC3, TSST-1) or *S. pyogenes* (SpeA and SpeC) have been engineered for high-affinity using yeast display and directed evolution. Here we review the engineering of these high-affinity Vβ proteins, structural features of the six different SAgs and the Vβ proteins, and the specific properties of the engineered Vβ regions that confer high-affinity and specificity for their SAg ligands.

## 1. Overview

Over the past 30 years, the family of exotoxins expressed by *S. aureus* and group A *Streptococcus* known as “superantigens” (SAgs) [1] has been studied extensively at the molecular and structural levels. There are 24 SAgs known to be expressed by *S. aureus*, and 11 SAgs known to be expressed by group A *Streptococcus* [2,3,4,5]. Despite their sequence diversity, these toxins exhibit a canonical structural motif that consists of two domains, a smaller N-terminal domain with two β-sheets and a larger C-terminal domain with a central α-helix and a five-stranded β-sheet [5,6,7,8]. This canonical structure has presumably allowed SAgs to maintain their ability to interact with a T cell receptor Vβ domain on one side of the molecule and a class II product of the MHC on another side [6]. This dual binding is required for activation of T cells and subsequent cytokine release, as monovalent binding of a ligand to the TCR is not sufficient for signaling. SAg-mediated crosslinking with MHC allows multiple MHC-bound SAg molecules to form a multivalent TCR complex, thereby initiating signaling [6,9,10]. 

The pathogenic function of SAgs is not clear, although it is likely related to their ability to dysregulate an immune response, or perhaps to generate a cytokine milieu that is favorable for survival of the organism. While the precise functional or evolutionary advantage of expressing a large family of SAgs with extensive sequence diversity is unclear, one clinical consequence has been that antibodies generated against one of the SAgs are not likely to cross-react with most of the other SAgs, thereby limiting the ability of an individual to neutralize multiple toxins [11]. Understanding the clinical correlates of SAg expression are further complicated because of the varied prevalence of individual SAg genes among different bacterial isolates, especially of *S. aureus* [12,13]. Most of the SAgs, including staphyloccocal enterotoxin A (SEA), SEB, SEC, and toxic shock syndrome toxin-1 (TSST-1), are encoded on variable genetic elements [14,15,16,17]. Thus, some strains express one or more SAgs while other strains can express a different pattern of SAgs. Finally, there is additional complexity because there is variation in SAg protein expression levels, with some evidence that SAgs SEB, SEC and TSST-1 may be expressed at higher levels than the other SAgs, due to transcriptional regulation [18].

Despite this variability in prevalence and expression levels, it is clear that the potency of SAgs is a direct cause of disease or at the least exacerbates a host of diseases. These include toxic shock syndrome (TSS), pneumonia, purpura fulminans, severe atopic dermatitis, and endocarditis [19,20,21,22,23,24,25]. While TSS is the disease most often associated with SAgs, especially TSST-1, the frequency of staphylococcal or streptococcal infections in specific tissues (e.g., lung, skin, soft tissue) results in SAg-mediated, hyper-inflammatory reactions at these sites [26,27]. Specific inhibition of such severe tissue inflammation could be a useful adjunct to treatment of these diseases. 

Given the considerable structural information about SAgs and their interaction with Vβ domains, we embarked over ten years ago on an effort to engineer soluble versions of the Vβ domains against various SAgs for the purpose of developing potent neutralizing agents that could suppress the hyper-inflammatory properties of SAgs [28]. A similar receptor-based strategy has worked for neutralizing the effects of TNF-α with the soluble TNF-α receptor/immunoglobulin fusion Etanercept (trade name Enbrel) [29,30]. Because of the low affinity of SAgs for their Vβ receptors, we reasoned that effective neutralization would require the generation of higher affinity variants of the Vβ, which would outcompete toxin engagement by TCRs bearing any Vβ region since the same binding epitope on the SAg is used regardless of the Vβ region expressed by the T cell. This affinity maturation has been accomplished using a directed evolution process and yeast display [31,32], an approach that has yielded, to date, high-affinity Vβ proteins against the six SAgs SEA, SEB, SEC3, TSST-1, SpeA, and SpeC [28,33,34,35,36,37]. Several of these have been used successfully in animal models of *S. aureus* infections involving (TSS), pneumonia, skin disease, and endocarditis [20,33,35,36,38]. Here we review features of the entire collection of high-affinity Vβ domains. 

## 2. Structural Features of the Superantigens

**Figure 1:**
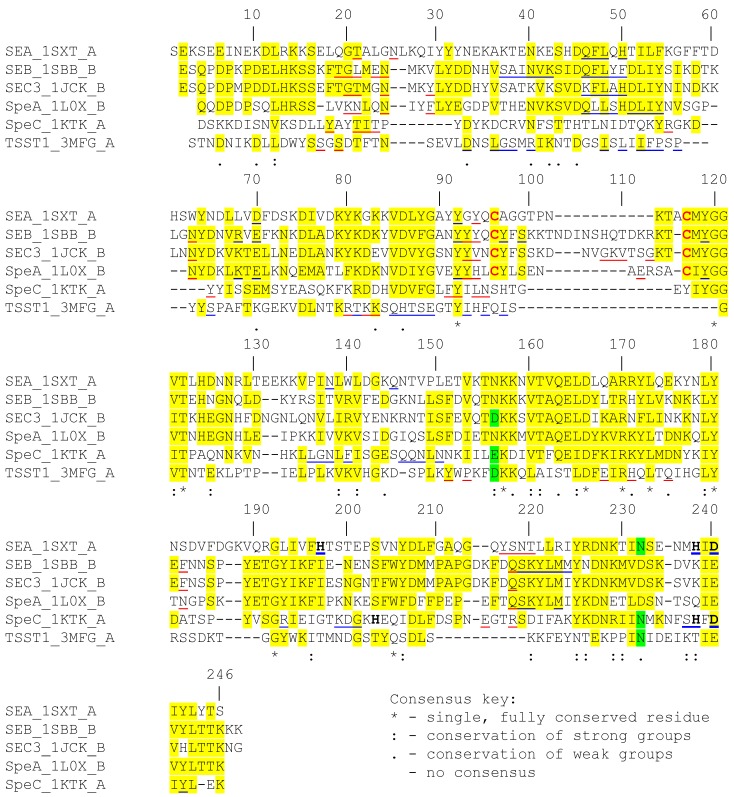
Sequence alignment of various superantigen sequences. The sequence of each superantigen (SAg) was obtained from the PDB file corresponding to its crystal structure. Multiple sequence alignment [CLUSTAL W (1.81)] was performed using “Biology WorkBench” online tool. Positions with homologous amino acids in three or more SAg sequences are highlighted in yellow or green. Residues in red are involved in forming the characteristic disulfide loop in certain SAg. Residues underlined in red and in blue are involved in binding to Vβ and class II MHC, respectively. Residues in bold are involved in binding zinc.

SAgs are structurally homologous, even though the primary sequences of the proteins are diverse [1,5]. These proteins are globular and range between 20 and 30 kilodaltons [5]. Although known staphylococcal and streptococcal SAgs have been classified into five groups based on differences in their amino acid sequences [5], here we focus on the six SAgs that have been the targets for high-affinity Vβ regions engineered by yeast display (see below). Sequence alignments of specific members of these groups (TSST-1 from Group I; SEB, SEC3 and SpeA from Group II; SEA from Group III and SpeC from Group IV) are shown in Figure 1. These six SAgs have 10% to 65% sequence identity among each other. SEB, SEC3 and SpeA are 50% to 65% identical, and perhaps it is not surprising that all three stimulate T cells with the same Vβ, mouse Vβ8.2 [5]. Although SpeC belongs to a different group than SEB, SEC3 and SpeA, it exhibits significant sequence homology with these proteins in specific regions, despite having overall low sequence identity (21% identity with SEB and SEC3, and 24% identity with SpeA). Overall, TSST-1 is the most distant and contains the lowest level of sequence homology (and 7% to 20% identity) to these SAgs.

Examination of the aligned sequences shows that there are several linear stretches of amino acids that are more similar among the six SAgs. Examination of their co-crystal structures with the variable regions of beta chain (Vβ) of TCRs or with class II MHC ligands suggest that these regions however are among the least homologous (Figure 1). These include residues near the N-terminus centered around position 20 which is part of the epitope for binding the Vβ region of the TCR [39,40,41,42]. Other Vβ binding regions are found between residues 90 to 95, and near the C-terminus of the protein sequences (positions 215–220), which also appear to lack the same degree of homology as flanking regions which are involved the structural framework for the SAgs. Thus, the residues in these regions, and also their atomic interactions with the cognate Vβ, most often differ and thereby account for the specificity of the interactions between SAg and the TCR. However, it is important to note that SAgs that employ a zinc-dependent binding site for interaction with class II MHC ligands use conserved residues for coordinating zinc ion (Figure 1).

SEA, SEB, SEC3 and SpeA also contain a homologous region (residues 45 to 55 in Figure 1) that serves as a binding site for class II MHC [43,44,45,46,47]. These same four SAgs, but not SpeC and TSST-1, possess a characteristic cystine loop of 9 to 19 residues [48,49], which has been implicated in emetic activity of SAgs. Toxins such as SpeC and TSST-1 that lack the cystine loop have been shown to exhibit reduced to no emetic activity [50].

**Figure 2:**
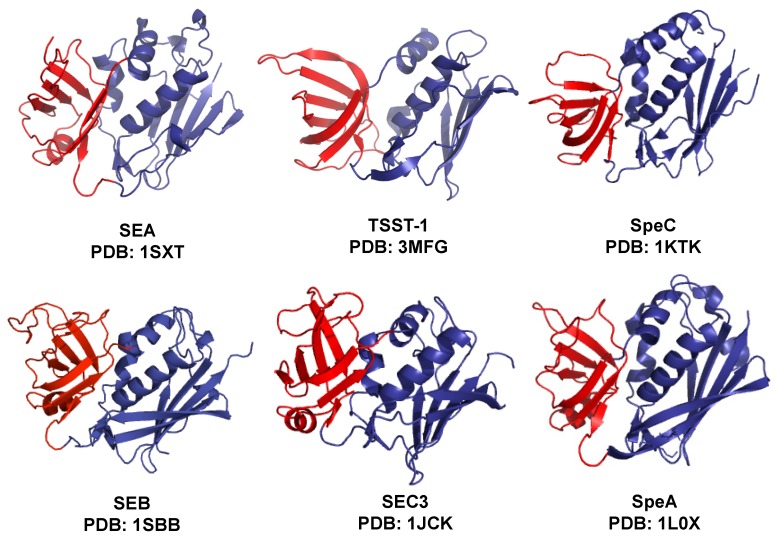
Two-domain architecture of superantigens. The canonical structure of SAg consists of two domains. The N-terminal domain (red) consists of a β barrel motif and C-terminal domain (blue) consists of a β-grasp motif and an α-helix which spans the center of the structure.

Although many of the staphylococcal and streptococcal SAgs possess overall low sequence identity, their structures possess striking similarity. The canonical structure consists of a N-terminal, β-barrel containing domain and a C-terminal domain containing a β-grasp motif and an α-helix which spans the center of the structure, connecting the two domains [5] (Figure 2). In the past two decades, a number of crystal structures of SAg have been solved (Table 1, only those that are relevant to this review are shown). Co-crystal structures with the Vβ region of TCR or class II MHC, have provided the basis for understanding SAg interactions with receptors on T cells and antigen presenting cells. The different modes of interaction of each SAg with these receptors reveal the diversity in mechanisms of binding to Vβ and MHC-II, which is particularly intriguing considering they possess highly conserved three-dimensional folds.

Four different modes of interaction of SAgs with class II MHC have been described: (1) SEB, SEC, SpeA bind to class II MHC alpha chain with a single, low affinity binding site that is located in the N-terminal domain of the protein. This binding is independent of the peptide located in the groove of the MHC-II molecule; (2) TSST-1 on the other hand uses a peptide-dependent binding mechanism to interact with low affinity to the class II MHC alpha chain through the TSST-1 N-terminal binding domain; (3) SpeC binds to the beta chain of class II MHC with high affinity, in a zinc-dependent manner through the C-terminal domain of SpeC; (4) SEA contains both a low affinity binding site and a high affinity, zinc-dependent site which could possible involve cross-linking of MHC molecules [43,44,45,47,51,52]. Structural features of SAg binding to the Vβ domains are described below.

**Table 1:** Crystal structures of staphylococcal and streptococcal superantigens and their complexes with Vβ domains.

Organism	SAg	Crystal Structure (PDB code, ligand)	Year	Reference
*S. aureus*	SEA	1ESF (co-crystallized with Cd^2+)^	1995	[53]
*S. aureus*		1SXT (co-crystallized with Zn^2+^)	1996	[54]
*S. aureus*	SEB	3SEB	1998	[55]
*S. aureus*		1SBB (co-crystallized with mVβ8.2)	1998	[39]
*S. aureus*		3R8B (co-crystallized with affinity matured mVβ8.2 mutant G5-8)	2011	[56]
*S. aureus*	SEC3	1CK1 (co-crystallized with Zn^2+^)	2002	[57]
*S. aureus*		1JCK (co-crystallized with mVβ8.2)	1996	[40]
*S. aureus*		2AQ3 (co-crystallized with affinity matured mVβ8.2 mutant L2CM)	2005	[58]
*S. aureus*	TSST1	2QIL	1996	[59]
*S. aureus*		2IJ0 (co-crystallized with affinity matured hVβ2.1 mutant D10)	2007	[42]
*S. aureus*		3MFG (co-crystallized with hVβ2.1 stabilized wild-type EP-8)	2011	[56]
*S. pyogenes*	SpeA	1FNU (co-crystallized with Cd^2+^)	2000	[60]
		1FNV (co-crystallized with Cd^2+^)		
		1FNW (co-crystallized with Cd^2+^)		
*S. pyogenes*		1L0X (co-crystallized with mVβ8.2)	2002	[41]
		1L0Y (co-crystallized with mVβ8.2 and Zn^2+^)		
*S. pyogenes*	SpeC	1AN8	1997	[61]
*S. pyogenes*		1KTK (co-crystallized with hVβ2.1)	2002	[41]

## 3. Engineering High-Affinity T Cell Receptor Vβ Domains against Superantigens SEA, SEB, SEC3, TSST-1, SpeA, and SpeC

Except for staphylococcal enterotoxin H (SEH), which has been shown to interact primarily with the variable region of alpha chain of the TCR [62,63], most SAgs are known to specifically interact with variable regions of TCR beta chain. The hallmark feature of SAgs is that they stimulate T-cells that bear a specific subset of variable regions in their beta chains (Vβ) [64,65]. Interestingly, despite their potent activity, SAgs are known to bind to their cognate Vβ receptors with low affinity (K_D_ values in the micromolar range) (Table 2 and [66,67,68]). In order to develop an antagonist that can effectively neutralize their toxic effects *in vivo*, a panel of six soluble, high-affinity TCR Vβ mutants have been engineered [28,33,34,35,36,37]. These Vβ mutants bind to one of six key staphylococcal and streptococcal SAgs (SEA, SEB, SEC3, TSST1, SpeA, and SpeC), at the same epitope as the wild type receptors but with much higher affinity, in the picomolar to nanomolar range. These represent 1000 to 3,000,000-fold increases in affinity, compared to wild-type (Table 2). Unlike antibodies, which could bind to any epitope of the SAg, engineering of the Vβ ensures that the neutralizing agent binds the identical SAg epitope as the wild-type receptor, thereby ensuring that direct competition and corresponding neutralization occurs.

**Table 2:** High-affinity Vβ domains that bind to various superantigens.

Organism	SAg	WT Vβ		High affinity Vβ		Improvement in affinity (fold)	References
		Name	Affinity (µM)	Name	Affinity (pM)		
*S. aureus*	SEA	Human Vβ22	100	FL	4,000	25,000	[37]
*S. aureus*	SEB	Mouse Vβ8.2	144	G5-8	50	2,880,000	[33,66]
*S. aureus*	SEC3	Mouse Vβ8.2	3	L3	3,000	1,000	[36,66]
*S. aureus*	TSST1	Human Vβ2.1	2.3	D10	180	13,000	[34]
*S. pyogenes*	SpeA	Mouse Vβ8.2	6	KKR	270	22,000	[35,66]
*S. pyogenes*	SpeC	Human Vβ2.1	20	HG_FI	500	40,000	[41,69]

**Figure 3:**
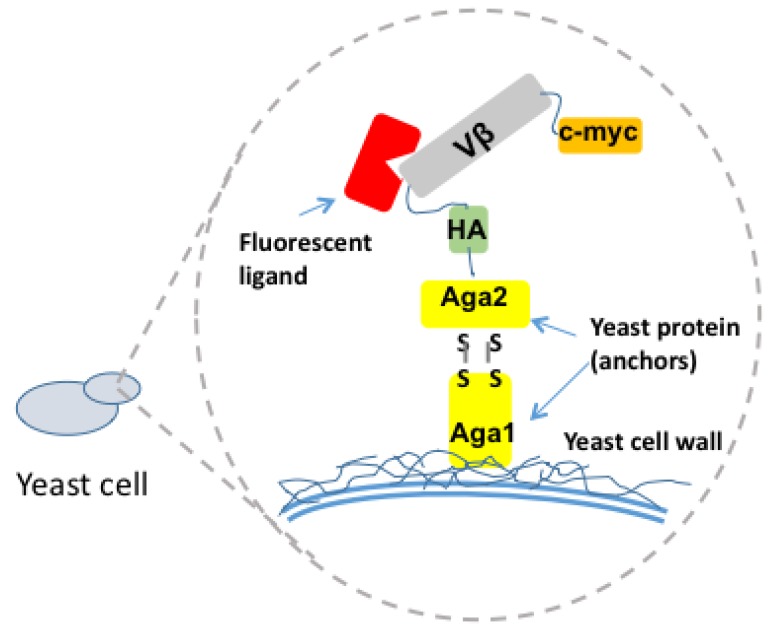
Schematic of yeast display system for engineering high-affinity Vβ domains against Superantigens. The Vβ libraries with various mutations are fused to the C terminus of the yeast mating protein Aga-2 to be displayed on the yeast cell surface. HA and c-myc tags are included in the fusion gene to probe and quantify the Vβ protein expression level. Fluorescent ligands include either a monoclonal antibody to the Vβ region or the SAg.

**Figure 4:**
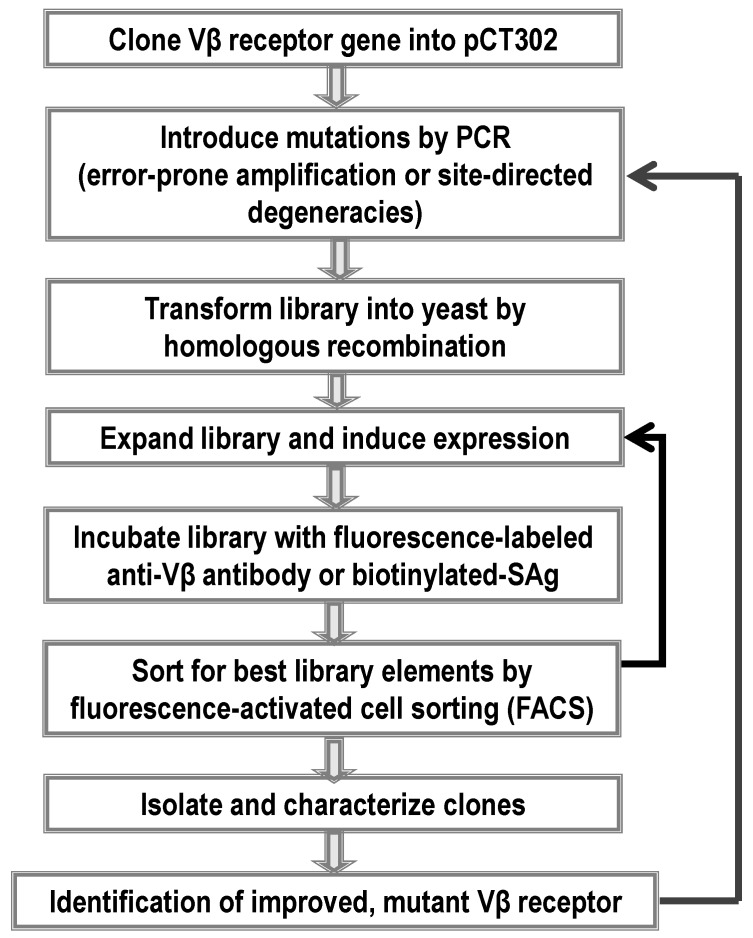
General flow chart for the cloning, display and engineering of high-affinity Vβ neutralizing agents by yeast display. A Vβ clone that is specific for the SAg of interest is cloned into the yeast display vector (Figure 3) and used to generate libraries of mutants. The libraries are selected with fluorescently-labeled ligands, (e.g., conformation-specific anti-Vβ antibodies or the SAg of interest). Multiple rounds of selections are conducted to enrich the Vβ mutants with desired properties, which serve as templates for subsequent library design and screening to achieve desirable stability or affinity of Vβ with SAg.

All high-affinity Vβ mutants were engineered using yeast display technology (Figure 3) [31,32] and directed evolution. The process involved use of a wild type Vβ from TCRs known to be stimulated by the SAg of interest. This Vβ gene was cloned into the yeast display vector in frame with the yeast mating protein, Aga2 to be displayed on the yeast surface. The Vβ gene was flanked by hemagglutinin (HA) and c-myc tags, which served as probes of the protein expression. Unlike many antibody variable domains, wild type Vβ domains typically require that one or more key mutations be engineered into the protein in order to be expressed on the yeast cell surface [70,71]. To accomplish this, the Vβ gene was subjected to error prone PCR to introduce random mutations, and the library was selected by fluorescence-activated cell sorting (FACS) with a conformation-specific anti-Vβ antibody (these are typically available commercially against most of the human Vβ regions). Anti-Vβ antibody is used, rather than fluorophore-labeled SAg at this stage, as the affinity of the SAgs with wild type Vβ are so low that detection by flow cytometry is not possible. The mutated Vβ region that allows it to be expressed on the surface of yeast is often called a stabilized Vβ as it has been shown that such mutations yield stabilized, soluble domains [72] (Figure 4).

Stabilized Vβ region genes served as templates for either additional random mutagenesis or for site-directed mutagenesis to generate libraries (Figure 4) with mutations in the putative SAg-binding sites. Typically, selection of the sites for mutagenesis was guided by the crystal structure of Vβ:SAg complexes. If structural information was not available, such as with the recent engineering of Vβ against SEA [37], the residues in CDR2 were chosen to generate the first generation site-directed mutagenesis libraries because of its central role in the interaction of other Vβ regions with SAgs, as observed in Vβ:SAg crystal structures [39,40,41,42]. To construct the site-directed mutagenesis libraries, amino acid positions were encoded by randomized codons (NNS) in primers, and cloned by PCR using overlapping primers. PCR products were transformed into yeast cells by homologous recombination, yielding library sizes of 10^6^–10^8^ transformants.

Pre-selected degenerate libraries typically exhibit no detectable binding with SAg by flow cytometry due to their low affinity, or loss of binding, by the great majority of mutants. To select for the rare Vβ mutants that exhibit higher affinity binding, libraries were subjected to several rounds of selection with a decreasing concentration of biotinylated-SAg, followed by staining with fluorescently-labeled streptavidin, and fluorescence-activated cell sorting (FACS). In each round of selection, a small fraction of cells that exhibited the top 1% fluorescence upon selection with SAg of interest, were collected and expanded for subsequent rounds of screening. When a distinct yeast population with positive SAg-staining emerged after 3~4 rounds of selection, yeast cells were plated and higher affinity clones were isolated, characterized and sequenced. In the recent engineering of Vβ22 against SEA, mutations isolated from the CDR2 library alone were capable of increasing the SEA affinity by 25,000-fold [37]. Even more strikingly, with the engineering of mouse Vβ8.2 against SEB, mutations from one CDR2 library accounted for a 220,000-fold increase of Vβ affinity with SEB (from 144 µM wide type affinity to 650 pM for G2-5) [33]. These results further validated the essential role of CDR2 loop in Vβ:SAg interactions (see below).

Following the initial selection, additional mutagenized libraries were often constructed in regions (CDR1, HV4 and FR3) that flank the CDR2 tertiary structure, using one or a combination of the first generation lead mutants as templates. After further affinity- or off-rate-based selections, mutants exhibited a more modest 10 to ~100 fold further increase in affinity. The specific region(s) where higher affinity mutations were successfully isolated for each pair of Vβ/SAg reflected, in part, the diverse binding modes of the SAgs with their cognate Vβs. Ultimately, mutations isolated from multiple libraries could be combined to generate the highest affinity mutants that yielded 1000 to 3,000,000-fold increases in affinity with targeted SAgs compared to the wild type Vβ.

## 4. Topology of Vβ:Superantigen Interactions

**Figure 5:**
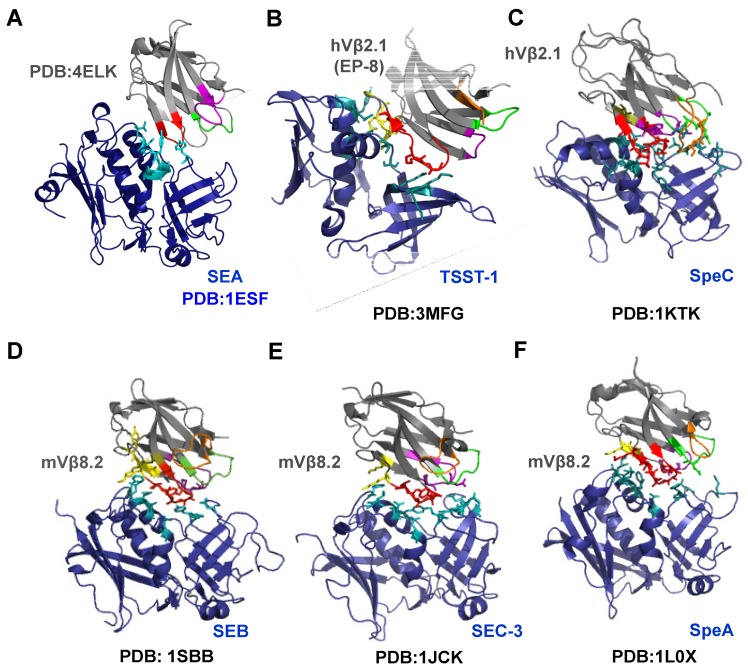
Co-crystal structures of six superantigens with cognate Vβ domain of the T cell receptor. Except for SEA, the co-crystal structures of SAg (blue) with their cognate Vβ ligand (gray) are available in PDB (Table 1). Residues of SAg interacting with Vβ are indicated in teal. Various regions of Vβ are colored as follows: CDR1 (green); CDR2 (Red); CDR3 (orange); HV4 (purple); FR2 (olive) and FR3 (yellow). Interacting residues of Vβ and SAg are displayed in stick configurations. (**A**) The SEA crystal structure was manually docked with mouse Vβ16 crystal structure (PDB: 4ELK), that is 66% identical to human Vβ22 protein sequence; (**B**) Co-crystal structure of TSST-1 with human Vβ2.1 mutant, EP-8; (**C**) Co-crystal structure of SpeC with human Vβ2.1; (**D**) Co-crystal structure of SEB with mouse Vβ8.2; (**E**) Co-crystal structure of SEC3 with mouse Vβ8.2; (**F**) Co-crystal structure of SpeA with mouse Vβ8.2.

Crystal structures of five out of six SAgs discussed in this review have been solved in complex with their cognate Vβ receptor ligand (Figure 5B–F) [39,40,41,42]. In general, the Vβ receptor docks in the cleft between the two domains of the SAg and uses its hypervariable loops (CDRs), or specific framework regions for engagement (Figure 5). Co-crystal structures of different SAg with their cognate Vβ ligands indicate that Vβ domains interact with the SAgs with considerable diversity in positioning and in interaction chemistries. However, the CDR2 loop of Vβ appears to be central to each SAg-TCR interaction [73,74] (Figure 5). Other regions of the Vβ appear to play important, but supporting roles, in the binding energy and specificity for the SAg [34,73,75].

SEB, SEC3 and SpeA (Group II SAg) are more structurally similar and each has been co-crystallized with murine Vβ8.2 (mVβ8.2) [39,40,41]. As indicated, these three SAgs possess 50%–65% sequence identity and they engage with the Vβ8.2 region of the TCR using similar residues (Figure 1), thereby determining their specificity for mVβ8.2. Accordingly, SEB, SEC3 and SpeA interact with mVβ8.2 with similar topologies (Figure 5), and they engage in intermolecular contacts primarily with CDR2 (accounting for 50%, 63% and 33% of total contacts respectively), HV4 and to some extent framework (FR) regions.

The mechanisms by which these three SAgs interact with mVβ8.2 are largely dependent on the common conformation of CDR2 and HV4, although SpeA forms a distinct contact via its E94 residue, by forming hydrogen bonds with N28 of CDR1 loop of mVβ8.2 [41]. Since SEB and SEC3 depend primarily on interactions with main chain atoms of Vβ-CDR2, their Vβ binding specificity is considerably reduced. However, SpeA:Vβ8.2 interaction specificity appears to be enhanced because the interface involves H-bonds between side chain atoms from both SpeA and the Vβ molecule.

In contrast, SpeC interacts with human Vβ2.1 (hVβ2.1) with more extensive use of the Vβ region, engaging all of the hypervariable loops. The specificity of the SpeC:hVβ2.1 interaction is increased by numerous H-bonds and van der Waals interactions with both main chain and side chain atoms of hVβ2.1. Non-canonical amino acid insertions in CDR1 and CDR2, and the presence of an extended CDR3 loop (at least in some β-chains), also increase the specificity of hVβ2.1 for SpeC [41].

Human Vβ2.1 is also the highly restricted target of TSST-1. Thus, both SpeC and TSST-1 interact with hVβ2.1 and both engage residues in CDR2 to make contacts with Vβ. Although the two toxins engage a few common residues in CDR2, each also uses other distinct, non-overlapping regions for binding and for achieving specificity. TSST-1 uniquely binds FR3 while SpeC engages with Vβ in a distinct mode by making extensive contacts involving residues from CDR1, HV4, FR2, FR3 and CDR3 in Vβ [41,42]. The specificity of TSST-1 for hVβ2.1 has been attributed to the involvement of hVβ2.1 FR3 residues E61 and K62. It has been speculated that TSST-1 does not activate T-cells bearing other Vβ domains because 75% of all other human TCR Vβ regions possess a proline at position 61, resulting in reduced conformational flexibility; this reduced flexibility could prevent the specific conformation required for interaction with TSST-1. In addition, the absence of a residue at position 62 in 50% of human TCR Vβ domains also contributes to the high specificity of TSST-1 for hVβ2.1 [42]. Finally, the residues that TSST-1 uses to interact with Vβ2.1 share little homology with residues that other SAgs (including SpeC) interact with their cognate Vβ ligand (Figure 1), which further enhances TSST-1 specificity towards hVβ2.1. Recently, the molecular basis of the extreme Vβ specificity of TSST-1 was determined to be the combination of both the non-canonical conformation adopted by CDR2 region of the Vβ along with residues Y56 and K62 on FR3 region [74]. 

## 5. Structural Basis of High-Affinity and Specificity of the Vβ:SAg Interactions

Although CDR2 regions have served as the predominant site for improving the affinities of Vβ domains for binding to their SAgs, it is clear that other regions can also serve to enhance affinity through structural changes in each Vβ:SAg interface. The involvement of regions other than CDR2, also can contribute to the high level of specificity in Vβ:SAg interactions exhibited by the high-affinity Vβ mutants. Several structures have been solved of SAgs in complex with engineered, high affinity Vβ domains, thereby providing an understanding of the interactions that confer both higher affinity and specificity [42,56,58]. Not surprisingly, multiple factors, including increases in van der Waals interactions, hydrogen bonds, hydrophobic interactions, cooperativity, and conformational flexibility have all been shown to be involved. Here we discuss the generation and structural basis of high affinity for select Vβ domains, which have been engineered over the past decade.

In order to generate a high affinity Vβ mutant for binding SEC3, mVβ8.2 was displayed on the surface of yeast and mutations were introduced by error-prone PCR, followed by site directed mutagenesis to combine mutations. One resulting mutant, called L2CM (K_D_ = 7 nM) (also called mL2.1/A52V, a first generation variant of L3, Table 1) exhibited ~450 fold increase in affinity compared to the wild type [28]. The structural basis of the SEC3:Vβ interaction has been studied extensively by alanine scan mutagenesis [76], and the high-affinity interaction with L2CM has been examined for binding energetics [77] and crystallization of various L2CM variants [58]. Although L2CM contained nine mutations, only four (A52V, S54N, K66E, Q72H) were energetically significant [77]. Structural analysis [58] indicated that the A52V mutation in CDR2, allowed an increase in hydrophobic contact area and also induced conformational changes in Q72 of the Vβ. The S54N mutation in CDR2 participated in affinity maturation by allowing recruitment of water molecules to SEC3:Vβ interface hence mediating contacts between N26^Vβ^ and N24^Vβ^ with D204, K205 and F206 in SEC3. Residue K66 appeared to be conformationally restrained in the SEC3:wtVβ8.2 structure, but it adopted a more extended conformation when mutated to glutamate. This also resulted in loss of van der Waals interactions with SEC3 and unfavorable change in enthalpy of binding but a highly favorable entropic change, resulting in a higher affinity complex [77]. Mutations Q72H and A52V were shown to be involved in inducing subtle conformational changes in hypervariable loops, thereby affecting how CDR1 residues, and CDR2 residue 52, interacted with SEC3. Although the A52V mutation had a dominant effect in affinity maturation by mediating restructuring events of the hypervariable loops, Q72H had a minor but significant contribution to affinity maturation [58]. Recently, L2CM was further engineered by incorporating additional mutations in CDR1, HV4 and framework regions to obtain mutant L3 (K_D_ = 3 nM) (Table 1 and [36]).

Similarly, mVβ8.2 was engineered for binding to SEB with a remarkable 3-million fold increase in affinity relative to wild-type mVβ8.2 (Table 1 and [33]). The engineered protein (G5-8) was crystallized in complex with SEB [56]. The structural details of this complex indicated that lengthening CDR1 loop by incorporation of a serine residue at CDR1 residue 27a, and incorporation of additional mutations N28Y and H29F, resulted in a distinct conformation of CDR1 loop. These mutations resulted in an increase in intermolecular contacts with SEB. Y28 in G5-8 was involved in pi stacking interaction with R110 in SEB and in H-bond interaction with N60. Additionally, two mutations (A52I and G53R) acquired in CDR2, resulted in replacement of residues with smaller side chains to relatively larger side chains which resulted in an increase in van der Waals contacts and H-bond formation with N31, N60 and N88 in SEB. Overall, it was concluded that an increase in the number of intermolecular contacts between G5-8 and SEB resulted in the significant increase in binding affinity [56].

Subsequently, mVβ8.2 was engineered for high affinity (K_D_ = 270 pM) for SpeA, using yeast display [35]. Key mutations, which were responsible for affinity maturation, were acquired in CDR2 (G53K, S54H), CDR1 (N30K) and in HV4 (Q72R). The resulting mutant, KKR showed a 22,000 fold affinity improvement compared to wt Vβ8.2 (K_D_ = 6 µM) [41]. Although there is no crystal structure of SpeA with the high affinity Vβ, the authors used revertant mutants and energy minimized computer modeling of the mutated Vβ -SpeA complex to propose the basis of this affinity maturation. The analysis revealed that the side chain of arginine acquired at position 53 in mutant KKR could be accommodated in a binding pocket in SpeA and promoted favorable interactions with side chain oxygen of Y90 and E88 of SpeA. Not only could this account for the affinity maturation, it could contribute to a lack of cross reactivity with SEC3, and the ability of mutant KKR to cross-react with SEB with high-affinity.

The mutant of hVβ2.1 called D10 was engineered for high affinity (K_D_ = 180 pM) against TSST-1, using yeast display (Table 1 and [34]). D10 contained 14 mutations relative to stabilized wt Vβ2.1 (EP-8). Of these 14 mutations, four were found to be energetically significant: three mutations in CDR2 (at residues 51, 52a and 53) and one mutation in FR3 (at residue 61). Surprisingly, positive cooperativity was observed between the distant mutations in CDR2 and FR3 [75]. Crystal structure analysis indicated that changes in intermolecular contacts, buried surface and/or shape complementarity were not the primary driving factor in affinity maturation of hVβ2.1 to TSST-1. Instead, altered conformational flexibility of D10 was proposed to have resulted in affinity increase by linking CDR2 and FR3 at the Vβ:SAg interface [42]. Using a similar approach, the hVβ2.1 region gene has also been engineered for high-affinity binding to SpeC (manuscript in preparation).

Finally, the hVβ22 region was engineered recently for high affinity binding (K_D_ = 4 nM) to SEA using yeast display [37]. The engineered mutant called FL contained ten mutations, of which five were located in CDR2. In the absence of a crystal structure of SEA with cognate Vβ, the authors suggested that the structural basis of high affinity may be improved electrostatic interactions (due to mutations N52E and E53D in CDR2), and pi stacking interactions involving N51Y in CDR2 with Y94 and Y205 in SEA’s putative TCR binding site.

## 6. High-Affinity Vβ Domains as Neutralizing Agents

The first study to validate the use of soluble, high-affinity Vβ domains in the neutralization of SAg activity was performed with the Vβ L2CM against the SAg SEC3. This work showed that soluble L2CM, but not soluble wild-type Vβ8, was able to completely inhibit SEC3-mediated T cell cytolysis at nanomolar concentrations [28]. This same high-affinity Vβ was fused to a class II MHC molecule in an attempt to increase the avidity of the interaction with SEC3 [78]. Although the fusion was shown to inhibit SEC3 activity *in vitro*, this study did not show whether the fusion had greater activity than the high-affinity Vβ alone. 

Subsequent studies showed that the high-affinity Vβ against SEB, G5-8, but not the wild-type Vβ8.2, was able to inhibit both *in vitro* and *in vivo* activities of SEB [33]. In this study, the *in vitro* activity was shown to be progressively improved in comparing different generations of mutants, with K_D_ values from 100 µM to 50 pM. For example, the 50 pM G5-8 protein was more effective at inhibition (*i.e.,* had a lower IC_50_) than the 650 pM G2-5 protein. Furthermore, the G5-8 protein was given to rabbits intravenously at the same time or after SEB administration, in an LPS-enhancement model of lethality, and the protein was able to prevent death even at concentrations close to stoichiometric with the SEB. In the same study, G5-8 administered daily to rabbits implanted with pumps containing SEB was able to prevent temperature increases and lethality due to the SAg. In a rabbit model of skin disease, G5-8 was able to inhibit the hypersensitivity reactions caused by SEB [38].

An in-frame fusion of the high-affinity G5-8 against SEB and the high-affinity D10 against TSST-1 yielded a single 30kDa protein, expressed in *E. coli*, was able to completely inhibit the *in vitro* activity of both SEB and TSST-1 [79], raising the possibility that multiple SAgs might be neutralized with a single therapeutic. An alternative approach is to identify a high-affinity Vβ domain that cross-reacts with multiple SAgs. While this has not been possible for structurally distinct SAgs (e.g., SEB and TSST-1), it has been shown that Vβ domains against SEB (e.g., G5-8) cross-reacted with high-affinity against SpeA, and that Vβ domains were capable of inhibiting both SEB and SpeA in the LPS enhancement models [35]. More recent findings showed that it is possible to engineer a cross-reactive neutralizing Vβ (L3) against both SEC3 and SEB [36].

The greatest clinical potential of soluble, high-affinity Vβ domains, aside from possible applications in biodefense, would be in serious diseases caused by *S. aureus*. The first study to show that high-affinity Vβ proteins were effective in diseases caused by *S. aureus* (*i.e.*, rather than the purified toxins), involved a rabbit model of pneumonia [20]. Rabbits receiving an intrabronchial inoculation (2 × 10^9^ cells) of *S. aureus* USA400 strain CA-MRSA c99-529 (SEB^+^) were protected from death when treated with 100 µg of G5-8, administered intravenously on a daily basis. Subsequent studies have shown that the high-affinity Vβ L3 against SEC3 also protected rabbits exposed to an SEC-positive strain of MRSA (USA400 MW2) in the pneumonia model [36]. Interestingly, the same L3 protein was capable of significantly reducing the bacterial burden of the MRSA (USA400 MW2) strain in an infective endocarditis model [36]. These pre-clinical studies suggest that these small Vβ proteins could be used intravenously, with antibiotics, to manage staphylococcal diseases that involve SAgs. The diversity of SAgs among different strains of *S. aureus* will likely require that diagnostics be developed for detection of the specific SAgs in patients, or that a multi-targeted therapeutic that can neutralize many of the SAgs be developed. 
